# Self-lubricating, low-friction, wear-resistant Al-based quasicrystalline coatings

**DOI:** 10.1080/14686996.2016.1152563

**Published:** 2016-03-15

**Authors:** Bruno Alessandro Silva Guedes de Lima, Rodinei Medeiros Gomes, Severino Jackson Guedes de Lima, Diana Dragoe, Marie-Geneviève Barthes-Labrousse, Richard Kouitat-Njiwa, Jean-Marie Dubois

**Affiliations:** ^a^ Laboratório de Solidificação Rápida, Universidade Federal da Paraíba, João Pessoa-PB, Brazil; ^b^ Institut de Chimie Moléculaire et des Matériaux (UMR 8182 CNRS- Université Paris-Sud 11), Bât. 410, 15 rue Georges Clémenceau, 91405Orsay Cedex, France; ^c^ Institut Jean Lamour (UMR 7198 CNRS – Université de Lorraine), Parc de Saurupt, CS50840, 54011Nancy, France

**Keywords:** Coatings, wear, friction, self-lubrication, quasicrystals, 10, 106, 206, 212, 306, 501, 504

## Abstract

After gas atomization, a quasicrystalline powder based on aluminium was used to prepare a thick coating by high-velocity oxygen-fuel flame torch spraying. This layer was deposited on top of a bond-coat layer on a steel plate. A post-spraying annealing treatment turned the two layers to their stable state, a γ-brass crystal and an icosahedral quasicrystal, respectively. The projection parameters were selected in such a way that the coating behaved like a self-lubricating material, which offered very good wear resistance (duration of pin-on-disk tests superior to 5 km with negligible material loss) and low friction (µ ≤ 6% against sintered tungsten carbide), in contrast to the state of the art. This property was achieved thanks to, on the one hand, excellent bonding to the substrate via the bound coat, and on the other hand, presence at the boundaries between quasicrystalline flakes of a mixture of both threefold and fourfold coordinated carbon originating from spray processing. Application to hard materials used in mechanical devices is appealing, especially because soft, lubricating additives may not be needed, thus considerably increasing the lifetime of the devices and reducing waste of materials.

## Graphical abstract

**Figure:**
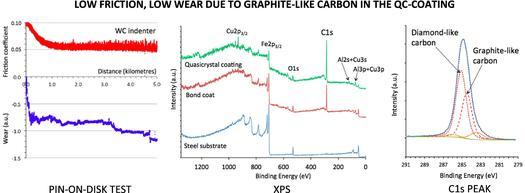

## 1. Introduction

**Figure 1.**
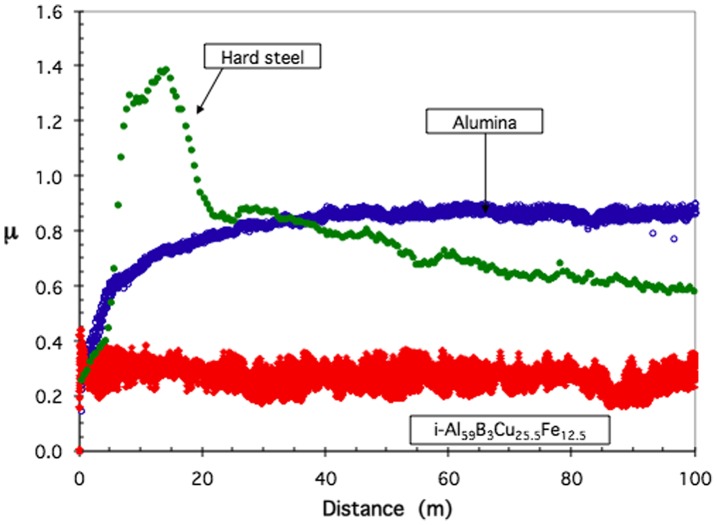
Summary of the state of the art friction against quasicrystals and more conventional hard materials as represented by the friction coefficient recorded as a function of sliding distance during pin-on-disk tests managed under a secondary vacuum of 10^−7^ mbar [3]. The materials under test are indicated in the figure: hard Cr-steel, sintered alumina and sintered quasicrystalline Al_59_B_3_Cu_25_Fe_12.5_ (at.%). The indenter was a Cr-steel sphere of 6 mm diameter. Other parameters were: load: 2 N, relative velocity: 5 10^−2^ m s^–1^. While Cr-steel sticks on itself, thus producing high friction and emission of abraded particles, it is progressively transferred onto the alumina material. Nothing like this occurs with the quasicrystal, which shows a comparatively moderate friction coefficient against hard steel.

Friction and resistance to wear are applied properties of key importance to mechanical engineering. Although these properties are not intrinsic to a material, since they depend on the nature of the antagonists, the geometry of the contact and its kinematics, and the possible presence of a third body, they have been the focus of many studies (for a review see [1, 2]). The force lying in the contact plane and opposing the sliding motion is known as the friction force (F_T_). The ratio μ = F_T_/F_N_, where F_N_ is the normal load, defines the friction coefficient. Macroscopic studies make use of a variety of experiments to assess friction between (in principle, well-defined) contacting solids. One method, called pin-on-disk, consists of a disk a few centimetres in diameter, which rotates in the horizontal plane at fixed velocity. A pin, or indenter, submitted to a normal load F_N_ of typically a few newtons, acts at a certain distance of the rotation axis. Friction produces a tangential force F_T_ that can be measured from the effort applied onto the pin holder. Examples of a spherical pin of Cr-steel sliding under a normal load of 2 N on the surface of three different materials are shown in Figure 1. They represent the state of the art concerning a quasicrystalline material of composition Al_59_B_3_Cu_25.5_Fe_12.5_ (at. %) compared to conventional materials such as hard Cr-steel and sintered alumina. Worth noticing is the very significant reduction of the friction coefficient measured on the quasicrystal in comparison to that observed on hard steel, despite the fact that they both exhibit similar high hardness and Young modulus. Those experiments however were conducted under high vacuum (typically 10^−7^ mbar) in order to limit oxidation of the wear debris that forms due to adhesion phenomena against the steel pin and contributes to a very significant extent to increase friction if the experiment is performed in ambient air or under oxygen. In this later case, friction of the quasicrystal against steel is typically as high as µ=0.6–0.8 whereas the duration of the test with no formation of wear debris is restricted to a fraction of a metre to few metres at most, depending on the sample microstructure and surface preparation. In contrast, in high vacuum, friction is found at a much lower level on a quasicrystal (Figure 1) as well as on a variety of Cu-containing complex intermetallics [4]. Friction is found to be higher if the composition of the crystal contains no Cu, but transition metals from the middle of the series. Dubois and Belin-Ferré [4] interpreted this effect as due to variations of the surface energy depending on the presence of d-states near the Fermi energy^[4]^.

Obtaining large friction coefficients is relevant whenever it is needed to transfer energy, for example with a train on its rails. The opposite situation is however quite frequent as well, but more difficult to manage practically. In developed countries, the waste of energy due to friction amounts to an appreciable few per cent of the gross national product, and intense research effort has been dedicated in recent years to overcoming friction losses in transportation industries, satellites, data storage hardware, etc. The use of liquid lubricants offers one solution to friction problems, but it is not perfect due to hydrodynamics and/or ageing of the liquid. Often, as in satellites or digital devices, liquid lubricants cannot be used. Solid lubricants, preferably incorporated in one of the contacting bodies, may offer a good alternative, but have their own limitations. For instance, easily delaminating materials such as MoS_2_ or graphite, soft metals such as tin, or polymers, can be used to produce self-lubricating composites that are able to exhibit friction coefficients against steel of the order of a few per cent or less. However, this is at the expense of a loss in hardness and toughness, which may lead to problems with the mechanical functionality of the device.

One potentially interesting solution to this dilemma is the use of a low friction material that is hard and tough, but also self-lubricating. Quasicrystals exhibit intrinsically low friction when sliding against hard steel (Figure 1) [3, 4], but unfortunately they are rather brittle at room temperature. Soon after a friction test is started in air, a third body forms from the worn out particles, which greatly increases the friction coefficient. This is a consequence of tribo-oxidation [5], which can be avoided only by placing the test bed in secondary vacuum [6]. Many attempts have been made to overcome this difficulty, for instance by enhancing the material’s toughness through inclusion of a soft metal in the quasicrystal matrix [7, 8] or by optimizing the preparation conditions of the friction counterpart [9]. The present article describes a self-lubricating quasicrystalline coating that shows friction against tungsten carbide not larger than 6% and of the order of 6–7% against hard steel, whereas wear resistance is excellent.

## 2. Experimental details

In this paper we report coating materials deposited on high-carbon 1010 steel plates of 5 mm thickness and few square centimetres in surface area. The Vickers hardness of this material is 170 HV_50_. The surface was prepared prior to coating by sand blasting and degreasing in acetone. Two coating layers were produced successively: first, a 200 µm thick bound coat of composition Al_4_Cu_9_ and second, a 110 µm layer of composition Al_59_B_3_Cu_25_Fe_13_, which was obtained from a gas atomized powder purchased from Saint Gobain Coating Solutions (Avignon, France) (powder ^®^F1). The preparation protocol of this powder was initially designed for plasma torch spraying, for production of low stick cookware on an industrial scale [10]. The Al_4_Cu_9_ material was prepared by alloying the raw elements in a levitation furnace under a protective argon atmosphere, then crushing pieces of the ingot in a planetary ball mill to produce a powder of adequate mesh size. This powder forms a γ-brass structure with 54 atoms per unit cell that was identified [11] as an efficient material to bond a quasicrystal to a metallic substrate, thus balancing the reduced adhesion towards metals characteristic of quasicrystalline alloys [3, 4].

We used a high-velocity oxygen-fuel (HVOF) flame torch to successively prepare the two coating layers. For the quasicrystal, the torch and spraying parameters (Table 1) were selected in order to set the flame on the reducing side, so as to avoid oxidation of aluminium during the time interval elapsing between melting the powder grains in the hot part of the flame and quenching them when they hit the steel plate, which was initially at room temperature.

**Table 1.** Processing parameters of the HVOF projection.

Gun model	P-HVOF Praxair JP-5000 Cannon: 4″
Oxygen flow rate	1.55 10^−2^ m^3^ s^–1^
Fuel	Kerosene
Fuel flow rate	4.42 10^−6^ m^3^ s^–1^
Flame velocity	Mach 3
Distance gun nozzle-plate	0.5 m
Relative velocity of gun versus plate	18.3 m s^–1^
Incidence angle of gun versus plate normal	0–20°
Powder feeding rate	0.013 10^−3^ kg s^–1^
Range of powder mesh size	25–45 µm
Initial plate temperature	14°C
Final plate temperature	120°C

Mechanical contact characterization was achieved using two different apparatuses, a scratch tester and a pin-on-disk from CSM Instruments, Peseux, Switzerland (now Anton-Paar). The indenter used for scratch testing was a Brinell sphere of 0.8 mm diameter made of diamond. Load was varied from 1 N up to 80 N and the indenter moved on the surface of the coating at a velocity of 10.5 mm min^–1^ over a total distance of 7.9 mm. Simultaneously, we recorded the noise emitting from the contact area. Optical and scanning electron microscopy observations were performed after the end of the test to assess the damage caused by the indenter. These observations were correlated to the two previous signals, based on the known displacement velocity of the indenter. Furthermore, we traced the penetration depth of the indenter by three-dimensional profilometry (3DP) and by analysing the nature of the chemical elements present in the scratch track with the help of conventional energy dispersive microanalysis (EDX). The pin-on-disk device was equipped with two different spherical pins, both of diameter 6 mm, which were charged with a constant load of 2 N and were actuated at a linear speed of 5.7 cm s^–1^. The tests were done in ambient air, at room temperature and relative humidity of 50%. We used a pin of cobalt sintered tungsten carbide and one of 100C6 hard Cr-steel (WC and HS, respectively, hereafter). Finally, we evaluated the hardness of the substrate and its two successive coatings by managing Berkovich nano-hardness measurements [12] on a polished slice of the sample cut perpendicular to its plane. The instrument was a NHT S/N 01-0100 nano-indenter that was monitored at a loading and unloading speed of 200 mN min^–1^ with a maximum load of 100 mN. We surveyed the chemical composition of all constituents of the sandwich by conventional energy dispersive analysis, using an EDAX PhiRhoz Quantification instrument (Mahwah, NJ, USA) equipped with SUTW Sapphire detector (Mahwah, NJ, USA) operated at 20 kV and a beam cross-section of approximately 1 µm.

Chemical analysis of the sandwich was performed by X-ray photoemission spectroscopy (XPS) on the outermost quasicrystalline coating surface as well as on a cross section cut perpendicular to the surface. The XPS spectrometer used (K Alpha, Thermo Fisher Scientific, EDAX Inc., Waltham, MA, USA, base pressure in the low 10^−9^ mbar range) was equipped with a monochromatic aluminium source (Al K_α_, 1486.7 eV). Spot sizes of 400 μm (corresponding to an irradiated zone of approximately 1 mm^2^) and 50 μm (corresponding to an irradiated zone of approximately 0.03 mm^2^) were used for surface and cross section analysis, respectively. The hemispherical analyser was operated at 0° take off angle in the constant analyser energy (CAE) mode, with a pass energy of 200 eV and a step of 1 eV for the acquisition of survey scans and a pass energy of 50 eV and a step of 0.1 eV for the acquisition of narrow windows. Charge compensation was done by means of a “dual beam” flood gun.

Prior to their introduction in the ultrahigh vacuum chamber, the specimens were ultrasonically cleaned using acetone, then ethanol baths. Ar^+^ ions sputtering at 500 eV and 6 × 10^−8^ mbar was used to remove the surface contamination layer in cycles alternating sputtering and data acquisition. Following each sputtering, survey spectra and C1s and O1s windows were recorded.

The recorded spectra were processed by means of the Avantage software (purchased from Thermo Fisher Scientific), using a peak-fitting routine with Shirley background and symmetrical 70–30% mixed Gaussian–Lorentzian peak shapes. The C/O ratio was evaluated following normalizations of the peak areas with the Scofield sensitivity factors.

## 3. Results and discussion

### 3.1. Sample characterization

**Figure 2.**
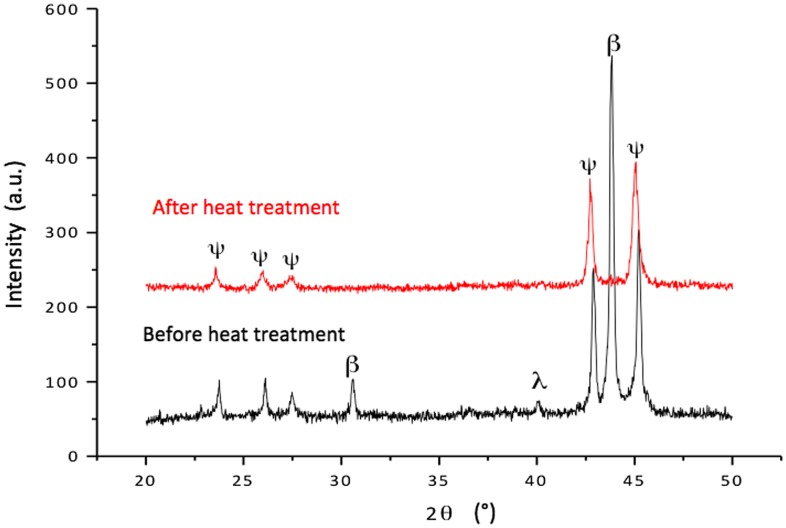
X-ray powder diffraction patterns (λ=KαCu=0.154184 nm) of the steel/Al_4_Cu_9_/Al_59_B_3_Cu_25_Fe_13_ sandwich at room temperature. X-rays investigate only the outmost side of the top layer. The lower pattern is for the sandwich before heat treatment as indicated in the text, and the upper pattern is for the same material but after heat treatment. Before heat-treating, the λ-Al_13_Fe_4_ and β-cubic metastable phases are clearly present, as a result of incomplete peritectic growth of the quasicrystal [10]; they have fully disappeared under the effect of the heat treatment. Observe the slight shift of the positions of the quasicrystal peaks due composition change when peritectic growth is completed.

Fast cooling associated with HVOF spraying traps the material in a metastable state. Growth of the quasicrystal is achieved through a peritectic reaction: liq.+β-cubic quasicrystal [13], which is a slow process. Obtaining a fully stable quasicrystalline state requires therefore a subsequent heat treatment, which we performed under argon atmosphere at a temperature of 730°C for 5 min [10]. Characterization by X-ray diffraction shows that the annealed samples are quasicrystalline (Figure 2). The upper side of the coated plate was then slightly polished down to a final arithmetic average roughness R_a_=9 ± 1 µm by mechanical grinding under water, avoiding any use of diamond paste or organic lubricant. The surface was degreased immediately before each test in a bath of acetone.

**Figure 3.**
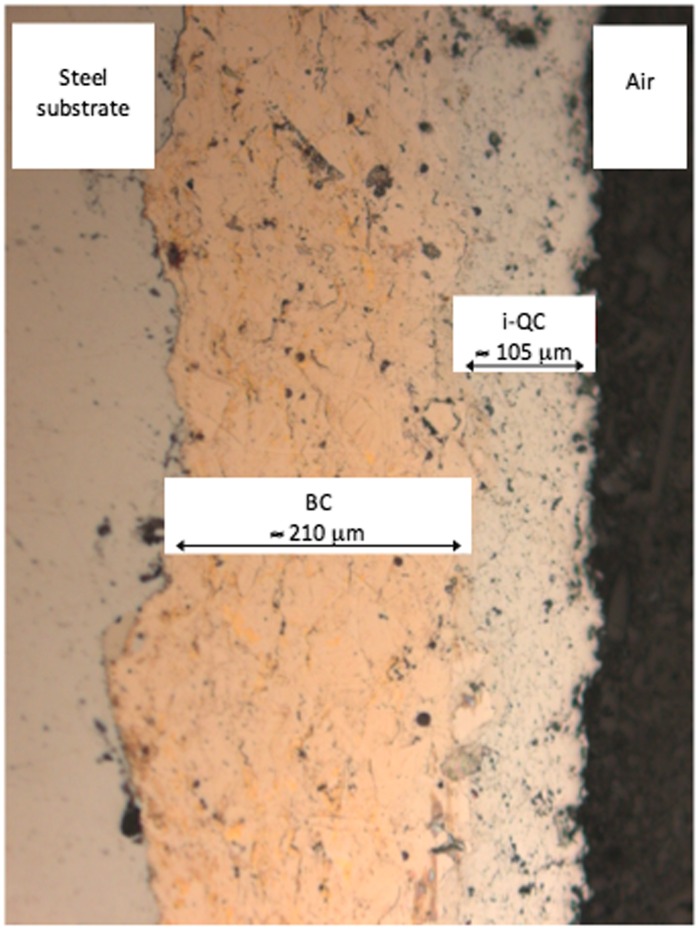
Optical micrograph of the steel/BC/i-QC sandwich sliced perpendicular to its plane. The steel substrate is placed on the left side of the figure and the top of the sandwich on the right. An approximate thickness of each layer is indicated.

**Figure 4.**
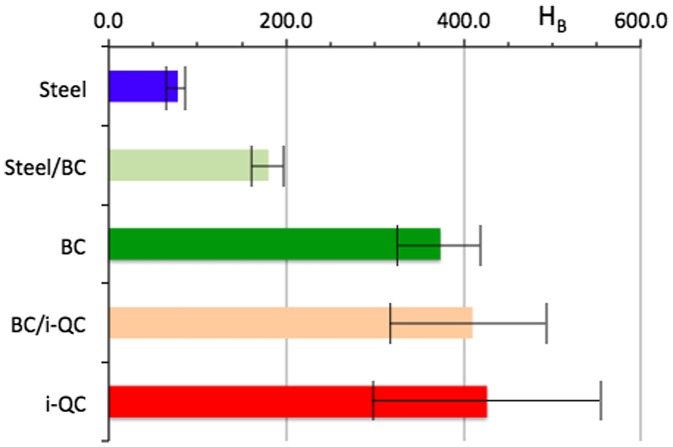
Graphical presentation of the Berkovich hardness H_B_ (load: 100 mN) as a function of position along a direction perpendicular to the sample plane. Error bars are taken equal to one mean square deviation among five measurements.

An optical micrograph of a steel/BC/i-QC (BC stands for bound coat and i-QC for icosahedral Al_59_B_3_Cu_25_Fe_13_ quasicrystal) is shown in Figure 3. Porosity is inevitable when using the HVOF technique, but this is reduced to a minimum. The interface between layers is sharp, thus emphasizing the adhesion between successive materials. As observed on the picture, the thickness of the BC layer is approximately 21 µm and that of i-QC around 105 µm. Using the Berkovich indenter, we measured the hardness of each component of the sandwich perpendicularly to its plane and monitored such measurements in, or very close to, each interfacial region. Results are summarized in Figure 4.

### 3.2. Scratch test

**Figure 5.**
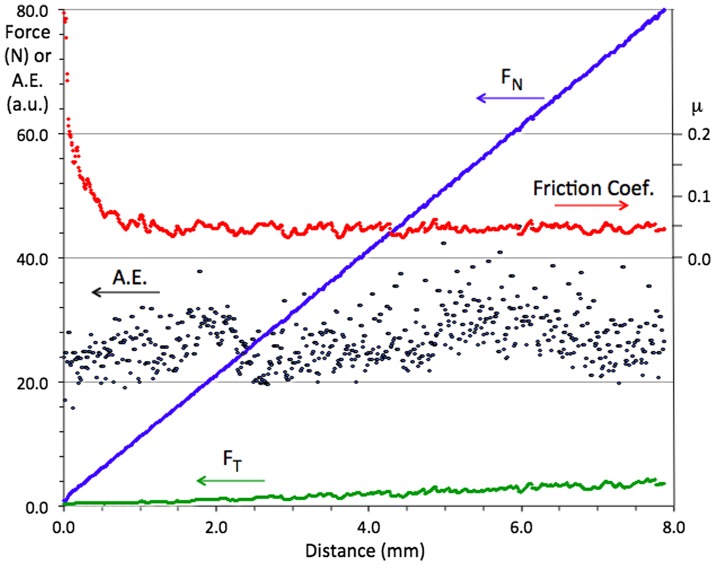
Typical scratch test chart recorded for the steel/BC/i-QC sandwich, using a diamond spherical indenter of 0.8 mm diameter. The normal load F_N_, tangential force F_T_, their ratio µ=F_T_/F_N_, and the simultaneous acoustic emission A.E. are shown.

A representative scratch test chart is shown in Figure 5. The material sample was the steel/BC/i-QC sandwich introduced above, and the indenter a diamond sphere of 0.8 mm in diameter. Load (F_N_) was increased linearly between 1 N (left side of the figure) and 80 N (right side of the figure) over a total distance of 7.9 mm and at a rate of 100 N min^–1^. The ratio of the tangential force (F_T_) over F_N_ defines the friction coefficient versus distance curve µ, which is shown amplified in Figure 5 to make it easier to read (right hand side axis). It decreases steeply in the beginning of the test, until all asperities of the surface are worn out, and then reaches a steady value equal to µ=0.046 ± 0.008, thus pointing out low friction in non-lubricated conditions. Furthermore, acoustic emission remains low during the whole duration of the test, which is a sign that small wear particles are emitted from the sliding track.

### 3.3. Pin-on disk tests

**Figure 6.**
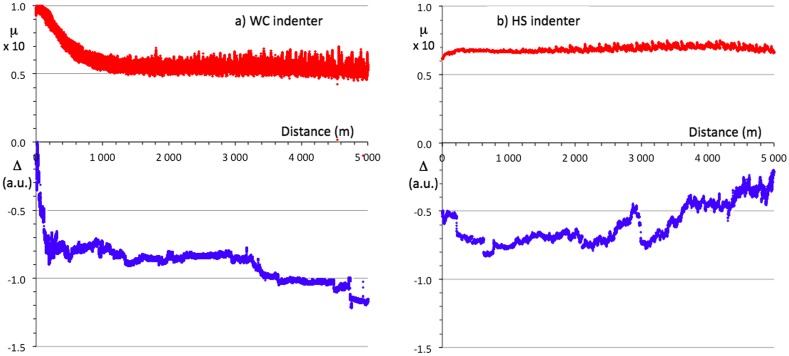
Pin-on-disk experiments on the steel/BC/i-QC sandwich using a sintered WC-Co indenter (a) and a 100C6 Cr-hard steel (b). The friction coefficient is represented by the upper curves and the change in vertical position of the sample surface-pin ensemble by the lower curve. Observe that both tests lasted for 5000 m.

Two pin-on-disk tests were produced on the steel/BC/i-QC sandwich, using a WC and a HS ball, respectively. Both indenters were 6 mm diameter spheres. The results are shown in Figure 6(a) and (b). Each test lasted for 5 km, which has not been achieved before with a quasicrystalline coating in ambient air, and is typically three orders of magnitude larger than the results of previous tests on quasicrystalline sintered materials or plasma sprayed coatings. In each figure, the upper trace is for the friction coefficient and the lower trace is for the position of the pin holder relative to an arbitration initial position. This measure, labelled Δ in the figures, represents in arbitrary units the wear experienced together by the two bodies in contact. For the WC counterpart, the steady state friction coefficient is µ=0.055 ± 0.008 whereas for hard steel, it comes to µ=0.07 ± 0.01. Except at the beginning of the test, when the initial roughness of the as-prepared surface is smoothed out, wear is not significant since the Δ values remain constant over large distances. Initial wear is due to the asperities left at the surface by the last step of polishing using corundum paper (so as to avoid organic lubricant of diamond paste being trapped in open pores). Sudden jumps of the Δ *vs* distance curve are merely due to mechanical clearance in the sample holder. In (a), the slight decrease of Δ with sliding distance signals the occurrence of a very low wear of either the pin or the sample (or both), whereas in (b), Δ increases, thus showing that worn out particles accumulate in the gap between pin and disk.

### 3.4. Chemical analysis

Inspection of the trace after the end of the test shows that no wear particles were emitted from the edges of the stripe. Plastic deformation was localized within the stripe and consisted essentially in cracks opened perpendicular to the direction of the indenter and plastic flow of the material (see the next sub-section). Energy dispersive analysis shows that Fe is present at the bottom of the scratch, even at the highest load of 80 N and far from the edges of the scratch. Since the indenter was a diamond tip, no transfer of iron from the indenter could occur, which proves that the diamond tip did not pierce the i-QC layer. In turn, the i-QC layer was not delaminated from its bound coat and wear was limited solely to the quasicrystalline layer.

**Figure 7.**
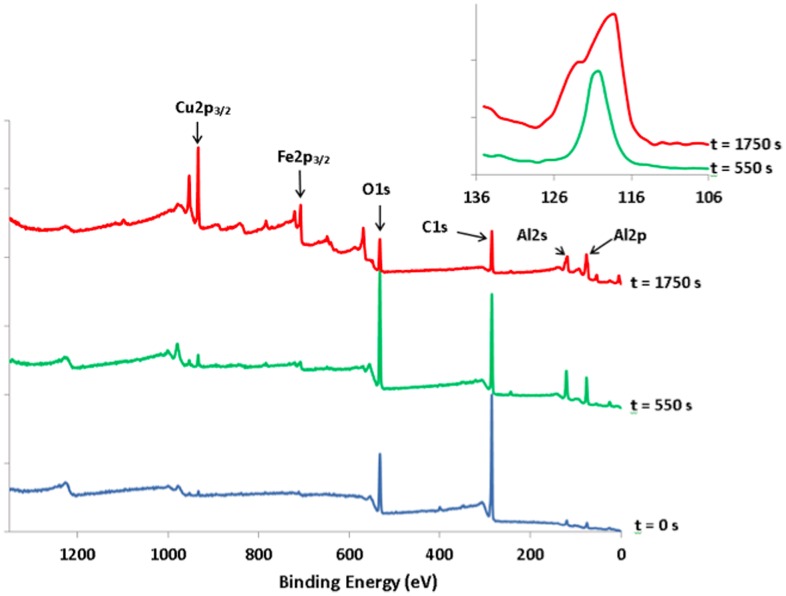
XPS survey data of the quasicrystalline coating surface before etching (t=0) and following 550 s and 1750 s of etching time with Ar^+^. Inset: enlargement of the Al2s-Cu3s region corresponding to 550 s (bottom plot) and 1750 s (upper plot) etching with Ar^+^.

In order to understand better why the sandwich studied here was far more resistant to wear than what was already published in the literature, we made a thorough study of the possible contamination layers that could lubricate the sample. Figure 7 shows survey data recorded on the outermost quasicrystalline coating surface before etching and following etching with Ar^+^ for 550 and 1750 s. On the as-received sample, large peaks corresponding to carbon and oxygen species are observed (atomic ratio deduced from the C1s and O1s intensities C/O=4), which can be ascribed to carbonaceous contamination, together with weak features corresponding to Al and Cu. Progressive etching of the surface leads to a decrease in the C1s signal and an increase in the Al, Cu and Fe signals which are well defined for Ar^+^ sputtering times longer than 950 s. The behaviour of the oxygen signals looks more surprising. Initial etching leads to an increase in the O1s peak (see data corresponding to 550 s etching in Figure 7) before it decreases following further etching. This behaviour can be explained by considering the presence of an oxide layer containing mainly aluminium oxide between the carbonaceous contamination layer and the quasicrystalline layer. Initial etching suppresses the carbon contamination and the signal mainly comes from the underlying oxide layer. Further etching progressively eliminates the oxide layer and peaks corresponding to the quasicrystalline coating are appearing. Further evidence of the presence of such an oxide layer can be deduced from a careful scrutiny of the Al2s region (see inset in Figure 7). The feature appearing in this region corresponds to the (overlapping) Al2s and Cu3s peaks. It has been shown by Wardé et al. [14, 15] that oxidation of the Al_4_Cu_9_ (110) surface leads to the appearance of a feature corresponding to oxidized aluminium located between the metallic Al2s (at 117.9 ± 0.2 eV) and Cu3s (at 122.9 ± 0.2 eV) peaks. The differences in the signal shape observed between data recorded following 550 s and 1750 s etching times are in good correlation with the decrease of the oxidized aluminium feature and the appearance of “metal” Al2s and Cu3s peaks.

**Figure 8.**
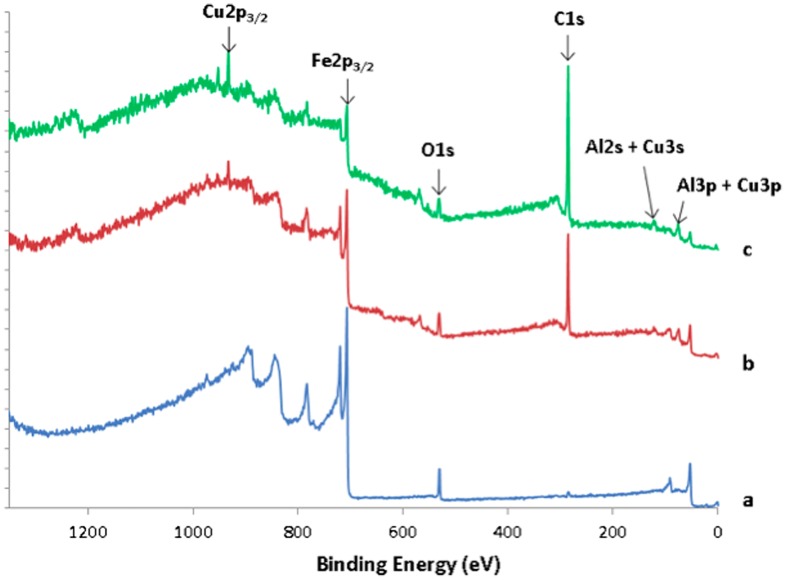
XPS survey data recorded on the transversal cross section of the sandwich, on the steel substrate (spectrum a), the bound coat (spectrum b) and on the quasicrystal layer region (spectrum c).

Figure 8 shows the survey spectra recorded on the transversal cross section of the sandwich, on the steel substrate (spectrum a), the bound coat (spectrum b) and on the quasicrystal layer region (spectrum c). It can be clearly seen from the C1s intensities that the quasicrystalline layer contains a much larger amount of carbon than the steel substrate. Except for the intensity of the C1s peak, survey of the BC layer does not show significant qualitative differences in comparison to spectrum c originating from the i-QC layer. This result indicates carbon originating from HVOF processing of the coatings. In this process, we used kerosene as a combustible, which may have supplied the carbon atoms found in the coating layers due to a specific choice of the parameters used for setting up the torch. More work is in progress to get insight into the detailed cracking mechanism of the kerosene molecules from which carbon may originate. It must be pointed out that, due to the poor lateral resolution of the technique, the XPS survey of the BC layer also contains contributions coming from the neighbouring layers. However, it can be seen from Figure 8 that the carbon concentration is higher in the quasicrystal layer compared to the BC one. It can be evaluated from the XPS survey spectra in a semi-quantitative manner, as for the ratio C/O. A ratio of 0.5 is obtained for steel, 8.7 for the BC layer and 14.8 for the i-QC layer.

**Figure 9.**
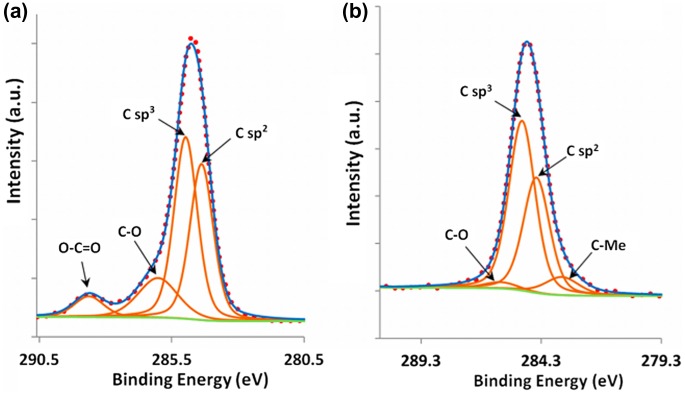
Peak fitting of the C1s feature (a) before etching and (b) following sputtering with Ar^+^ for 1750 s.

In an attempt to understand the nature of carbon contained in the coating, a decomposition of the C1s spectra obtained before and after etching the quasicrystalline coating topmost surface was performed. The results of the decomposition are presented in Figure 9(a) and (b) for the as-received sample and after 1750 s etching, respectively. Five contributions were necessary to achieve good peak fitting, which can be ascribed to the two hybridization states, sp^2^ at 284.3 eV and sp^3^ at 284.8 eV, the C-O bonds (formed either with surface oxygen or hydroxyl type bonds) at 286 eV and C=O (carbonates) at 288.6 eV and C-Me (Me=metal) at 283.4 eV. The precision in the peaks binding energy is ± 0.2 eV. These values are in good agreement with the literature [16–18]. The most striking feature in Figure 9(b) is the presence in the quasicrystalline layer of a substantial amount of carbon, both graphitic and diamond-like (with a sp^2^/sp^3^ ratio approximately equal to 0.7), which cannot be ascribed to carbonaceous contamination in view of the very small amount of residual C-O species. We assign the origin of this carbon to the HVOF process during preparation of the bound coat and quasicrystalline layer. Further work is in progress to better understand this point.

### 3.5. Abrasion and wear

The experimental investigations by the scratch test and by the pin-on-disk test revealed that the material under study has excellent wear resistant properties. With regard to the scratch experiment, a shallow residual depth was observed immediately after the end of the test by backward probing of the track without moving the sample. As usual, we attempted to extract a profile of the track using a conventional profilometer. The operation was unsuccessful because it was almost impossible to differentiate the scratch track from the surface roughness. This means that the deformation of the sample is mainly elastic. Thus, up to a high load level (80 N), plastic deformation of the sample is low and is confined at the bottom of track within the upper (i-QC) layer (see section above). The inability of the material to conform to the groove is indicated by the conformal micro-cracks within the track. Let us now consider the case of the wear test performed on a pin-on-disk device. As can be observed in Figure 6a for the WC indenter, the wear produced after a long distance of sliding is very low. One outstanding fact is that practically no wear is produced during a long sliding distance of few thousands metres. The associated friction coefficient is also unusually low, almost half the values usually presented in the literature; e.g. [19]. However, many factors can affect the tribology of contacting bodies under relative sliding motion. In the present case, the performance of the material may be attributed to the presence of sp^2^-type carbon in the layer (see section above). Indeed, it has been shown for example the addition of carbon in P/M FeAl alloy greatly improves the wear resistance, an effect attributed to the lubrication characteristics of graphite [20]. This lubricating effect of carbon is probably responsible for the lowering of the friction coefficient in the case of the HS indenter, which is softer than the WC. Despite this low friction coefficient, a surprising effect is observed. The relative position of the pin on the counterpart surface varies in the wrong way, at least after a distance of 2000 m (Figure 6(b)). This indicates that there is a third body, which builds up in the contact area and is not due to the analysed material. An inspection of the worn surfaces revealed that the third body within the track comes from the wear of the pin. We assign this effect to the presence of sp^3^-carbon, which produces wear on the non-lubricated counterpart. A better understanding of this abrasive property of the analysed material requires further investigation.

## 4. Conclusions

We found that HVOF is suitable to produce fully quasicrystalline coatings, free of any crystalline residual phases after heat treatment. They highly adhere to a carefully selected bound coat like γ-Al_4_Cu_9_, hence to the substrate. The friction coefficient against diamond (µ=0.05 in linear scratch testing) is very significantly smaller than that of steel sliding against itself. Severe scratch tests up to a normal load of 80 N, using a diamond spherical indenter of 0.8 mm diameter, or long duration pin-on-disk tests under 2 N load using a Cr-100C6 spherical indenter of 6 mm diameter, deform the quasicrystalline layer significantly, but not to an extent that would be sufficient to pierce that layer, thus emphasizing the self-lubricating capacity of the coating. Self-lubrication is assigned to the presence within the layer of substantial amounts of carbon, both of graphite and of diamond type, that are produced by the HVOF process itself. HVOF-prepared quasicrystalline coatings therefore appear as excellent potential candidates for surface reinforcement of parts submitted to intense friction against hard steel, as e.g. in non-lubricated gear boxes, and especially when operating in dry conditions or in vacuum. More detailed results on a variety of substrates and operating conditions regarding experimental as well as computed mechanical performance of the coatings will be presented elsewhere.

## Competing interests

To the best knowledge of the authors, there is no competing interest involved in the present study.

## Authors’ contributions

BASG prepared the samples and produced the experimental data under the supervision of RMG and SJG in Brazil and RKN in France. DD and MGBL did the XPS experiments and interpreted the data. JMD designed the study and wrote the manuscript. All authors took part in the discussions and contributed amendments to the manuscript. They all agreed to co-author this article.

## Disclosure statement

No potential conflict of interest was reported by the authors.
